# Mastitis and Its Treatment

**Published:** 1867-05

**Authors:** Robert S. Addison

**Affiliations:** 269 Milwaukee Avenue, Chicago


ARTICLE XXII.

### MASTITIS AND ITS TREATMENT.

BY ROBERT S. ADDISON, M.D., 269 Milwaukee Avenue, Chicago.

As it has at this day become very *fashionable*—I believe that is the right word to apply—for mothers to wean their children early; if, indeed, they nurse them at their own breasts at all, our attention has been, lately, more frequently directed to the treatment of mastits and mammary abscess than formerly.

Regarding mastitis, in most instances we are not called upon to render relief until the breast has become painfully distended, and we find our treatment (which is directed mainly to the prevention of abscess,) of no avail, in a great many cases.

Having, in my own practice, had this occur so often, after having made use of cathartics and alterants, and relying much upon the liniments and ointments, hitherto so much extolled, without obtaining the desired and anticipated results, I have adopted a different mode of treatment, and one which has given me more gratifying results than those previously employed.

I believe that the effects of liniments and ointments are counteracted by the friction employed in their application, and now never make use of them, but proceed as follows:

If called before resolution has commenced, I begin with a saline cathartic, and direct my patient to avoid the use of fluid, as much as possible, and vegetables entirely, and make use of a diet composed of bread and meat, taken as dry as is endurable.

In many instances, this general treatment alone is sufficient to relieve the mastitis.

If, however, the breast has become painfully distended before I see the case, I supplement the foregoing treatment, by having an ordinary glass breast tube heated by immersing it for awhile in hot water, then, tightly closing the small orifice and cooling the large one until it can be comfortably borne upon the breast apply it over the nipple; then cover the tube with a cloth wrung out of cold water; and thus obtain easy and regular suction, which—repeated if necessary—entirely relieves the over-distended breast.

Where I find the patient in humble circumstances, I direct the use of a common beer-bottle instead of an ordinary breast tube; but it is not so well borne, owing to the smallness of the orifice and the irregularity of the surface which comes in contact with the tender areola.

*March 14th, 1867.*

